# Experimental assessment of *Acanthopagrus schlegelii* biomass based on environmental DNA technology

**DOI:** 10.1038/s41598-024-83590-2

**Published:** 2024-12-30

**Authors:** Yan Liu, Mengyi Zhang, Liangming Wang, Changping Yang, Yukai Yang, Qijian Xie, Manting Liu, Cheng Chen, Chunbin Jia, Binbin Shan

**Affiliations:** 1 South China Sea Fisheries Research Institute, Chinese Academy of Fisheries Sciences, Guangzhou, 510300 China; 2 Key Laboratory of Marine Ranching, Ministry of Agriculture Rural Affairs, Guangzhou, 510300 China; 3 Shenzhen Fisheries Development Research Center, Shenzhen, 518067 China

**Keywords:** *Acanthopagrus schlegelii*, Environmental DNA, qPCR, Marine biological monitoring, Fishery resources, Ocean sciences, Biodiversity

## Abstract

The Environmental DNA (eDNA) technology has attracted significant attention due to its convenience and high sensitivity. However, the variations of eDNA across diverse environments and biological species remain complex. Therefore, a detailed exploration of the release patterns of eDNA for specific species under different environments is crucial for the scientific utilization of eDNA detection techniques. This study conducted an experiment involving the aquaculture of *Acanthopagrus schlegelii* to explore the release and degradation mechanisms of eDNA. It also analyzed the influence of salinity and biomass on the concentration of eDNA in water. Through model simulations, the variation patterns of *A. schlegelii* eDNA were revealed. The study achieved three key findings: (1) The research on the release and degradation mechanisms of *A. schlegelii* eDNA indicated that the Generalized Additive Model (GAM) effectively fits the variation patterns of eDNA concentration. The peak concentration of eDNA released by *A. schlegelii* was observed at 42 h, and the degradation process exhibited two stages: rapid and slow degradation, with a negative correlation between eDNA concentration and time. (2) By investigating the relationship between the concentration of *A. schlegelii* eDNA and biomass, it was demonstrated that Linear Models (LM) effectively captured this relationship, indicating a correlation between eDNA concentration and biomass. (3) The detection of *A. schlegelii* eDNA concentration under different salinity conditions revealed that the GAM model better reflected the relationship between eDNA and salinity, exhibiting a negative correlation. As salinity increased, the concentration of eDNA decreased. This study lays a foundation for future assessments of the *A. schlegelii* biomass in natural waters using eDNA quantitative detection techniques, and provides relevant references for quantitative eDNA detection techniques in other marine fish species.

**Supplementary Information:**

The online version contains supplementary material available at 10.1038/s41598-024-83590-2.

## Introduction

Due to the impacts of habitat degradation, environmental pollution, and overfishing, marine fishery resources in China have been continually declining^1^. To address this challenge, researchers and managers worldwide have started to actively promote stock enhancement activities to restore the sustainability of fishery resources^2^. With the implementation of these activities, subsequent resource surveys and evaluations of stock enhancement effects have been carried out^3^. However, traditional survey methods such as direct observation, trawling, and acoustic measurements, while providing fundamental data for fishery management, have limitations in terms of cost, time efficiency, and potential environmental disturbances^4,5^. These methods are particularly inefficient in tracking low-density species and assessing long-term population changes^6^. Therefore, there is an urgent need to develop more scientific, efficient, and accurate assessment methods to advance the sustainable management and protection of fishery resources.

With continual advancements in molecular biology and ecology, environmental DNA (eDNA) technologies have become a revolutionary tool for monitoring biodiversity. eDNA refers to traces of DNA that organisms leave in their living environments^7,8^. Researchers can detect species present in specific aquatic environments by analyzing water samples for eDNA^7,9^. Due to its convenience, high sensitivity, and independence from temporal and weather conditions, eDNA technology has been widely applied in aquatic biological monitoring^10^. Particularly in the realm of biomonitoring, eDNA technology, with its nondestructive and high-throughput sequencing capabilities, offers an effective method for the early detection and monitoring of low-density populations^11,12^, endangered species^13–15^, and invasive species^16–19^.

However, eDNA technology is susceptible to numerous environmental factors, which often lead to biases in biomass assessments^20–23^. Additionally, eDNA release patterns vary among species, and the patterns of eDNA fluctuations vary significantly under different conditions^24–26^. Previous research has demonstrated that in small water environments such as experimental aquaria, ponds, and streams, there is typically a linear relationship between eDNA concentration and fish biomass, suggesting that analyzing eDNA concentrations in water samples can estimate the biomass of specific fish species^27^. However, this linear relationship becomes more complex and uncertain in larger water environments such as large rivers and other water bodies^28^. The environmental variability and ecological complexity are notably greater in large water environments, influenced by multiple factors such as tides, ocean currents, microbial activity, and light conditions, which may affect the distribution, dilution, and degradation processes of eDNA^10,11,29,30^. Therefore, it is essential to conduct detailed investigations into the eDNA release patterns of specific species under different environmental conditions to scientifically utilize eDNA detection technology.

First, the biomass of marine fish may directly influence the concentration of eDNA^31^. For instance, Jo et al. (2019) reported that in environments with relatively high water temperatures and large populations of *Trachurus japonicus*, the degradation rate of eDNA notably accelerated, suggesting a potential correlation between eDNA degradation rates and the biomass of this fish species^32^. Furthermore, the release and degradation processes of eDNA are also focal points of research^33^. This includes analyzing the stages from eDNA release from organisms to complete degradation, as well as the effects of temporal changes on these processes^33^. Minamoto et al. (2017) and Strickler et al. (2015) revealed that the rate of fish eDNA release is linked to the water residence time and degradation rate^24,34^. Moreover, salinity, a fundamental characteristic of marine environments, may affect the stability and degradation rate of eDNA, necessitating an exploration of its specific impact mechanisms^35^. Research by Saito and Doi (2021) indicated that at relatively high salinities, the degradation rate of eDNA decreases, thereby prolonging its persistence in aquatic environments, which may suggest a positive correlation with salinity^26^. By investigating the patterns of eDNA variation under different conditions, eDNA technology can be more accurately utilized to monitor and assess the biomass of marine fish, thereby providing a scientific basis for marine ecological protection and resource management.

*Acanthopagrus schlegelii* (Bleeker 1854) belongs to the Sparidae family and the genus *Acanthopagrus*^36^. Its unique ecological traits and wide distribution make it a significant economic fish species in China’s coastal regions^37^. *A. schlegelii* is widely favored for its rapid growth rate, robust disease resistance, and delicate flesh quality^38^, making it an ideal candidate for stock enhancement activities^39^. Therefore, accurately assessing the biomass of *A. schlegelii* is crucial for implementing effective fisheries management and resource conservation measures^1^. This assessment aids in understanding the health and trends of the *A. schlegelii* population and provides a foundation for the sustainable utilization of its resources.

This study investigated *A. schlegelii* through aquaculture experiments and explored the mechanisms of eDNA release and degradation, as well as the concentrations of *A. schlegelii* eDNA under different salinity and biomass conditions. By fitting the models to these conditions, we delineated the patterns of eDNA changes. These findings lay a foundation for future developments in biomass estimation within natural marine environments using quantitative eDNA detection techniques. This research provides a reference for further studies on the use of eDNA quantitative analysis techniques in other marine fish species.

## Materials and methods

### Experimental design

#### Influence of salinity on the eDNA concentration

This study investigated the impact of salinity on the concentration of eDNA from *A. schlegelii* through controlled aquaculture experiments. Four experimental groups were established, each containing 200 L of water with salinity gradients of 5, 15, 25, and 35. Each group had three replicates, along with a blank control group for comparison. All groups were stocked with five similarly sized *A. schlegelii* (approximately 660 ± 20 g per tank). Then, 1 L water sample was taken from each group for filtration to analyze the eDNA concentration.

#### Mechanisms of eDNA release and degradation

This study explored the release and degradation mechanisms of *A. schlegelii* eDNA through aquaculture experiments. The experimental design included three parallel control groups, each containing five similarly sized *A. schlegelii* (approximately 597 ± 10 g per group), and one blank control group, with each group’s water volume set at 330 L. Water samples of 1 L were filtered for eDNA analysis at 0, 6, 18, 42, 66, 90, 114, and 138 h. Following the release experiment, the *A. schlegelii* were removed to initiate the degradation experiment, with the timeline reset and water samples of 1 L each filtered for eDNA analysis at 3, 6, 12, 24, 36, 48, 72, 96, and 120 h.

#### Relationships between biomass and eDNA concentration

This study explored the relationship between the concentration of eDNA and the biomass of *A. schlegelii*. The experiment was arranged into five groups, each with 330 L of water containing 1 (0.13 kg), 2 (0.24 kg), 4 (0.52 kg), 8 (0.92 kg), or 16 (1.81 kg) fish. Additionally, five parallel control groups and one blank control were established. The experiment lasted for 72 h, after which a 1 L water sample was filtered from each group to analyze the eDNA concentration.

### Experimental setup

The *A. schlegelii* aquaculture experiment was conducted at the Shenzhen Experimental Base of the South China Sea Fisheries Research Institute, Chinese Academy of Fishery Sciences. The aquaculture water was sourced from the Dapeng Sea area in Shenzhen. Upon arrival at the experimental site, *A. schlegelii* were acclimated in the aquaculture ponds for three days to adapt to the new environment. Prior to the experiments, the seawater was filtered through a fabric filter to remove impurities. For each experimental group, precise measurements of *A. schlegelii* body length and weight were taken, and precautions were taken to avoid injuries during weighing. The experiment utilized a recirculation system with live fish and replacement of any deceased specimens. Water parameters in each aquaculture tank, including temperature, salinity, pH, and dissolved oxygen content, were measured and meticulously recorded alongside observations of *A. schlegelii* conditions, such as activity level, feeding behavior, and any abnormal behavior. Additionally, regular feeding and removal of excess feed were conducted to maintain stable water quality, providing a controlled environment for the study of eDNA dynamics.

### Water sampling and filtration

To ensure minimal contamination during sampling and filtration, laboratory personnel strictly wore work attire and disposable gloves. After collection, the sample bottles were cleaned with 0.1% sodium hypochlorite. This study utilized a portable water sampling filtration system (Tianjin JinTeng Experiment Equipment Co., Ltd.), which included GM-0.33 A Diaphragm Vacuum Pump, tubing, and a filtration flask. Tools were thoroughly disinfected before each filtration process. Each filtration was conducted in a well-ventilated space free of aquatic life, and purified water was used as a negative control before each filtration. Mixed cellulose ester (MCE) membranes with a pore size of 0.45 μm were used to avoid the clogging and degradation of eDNA, with the number of membranes used per filtration varying based on the turbidity and filtering difficulty of the water samples. The number of membranes used for each sample filtration is approximately 1–4. After filtration, the membranes were stored in cryogenic tubes, initially placed in liquid nitrogen, and later transferred to an ultralow temperature freezer for preservation.

A total of 134 water samples were collected. Of these, 40 samples were used to investigate the dynamics of eDNA release from *A. schlegelii*, 45 samples were utilized to explore the degradation dynamics of *A. schlegelii* eDNA, 17 samples were collected to examine the impact of salinity on the concentration of *A. schlegelii* eDNA, and 32 samples were used to study the relationship between biomass and the concentration of *A. schlegelii* eDNA.

### eDNA extraction

DNA was extracted using the DNeasy Blood and Tissue Kit (Qiagen, Germany). The protocol was modified by adjusting the concentrations of the first two lysis buffers and the protease to ten times the standard concentration to enhance the lysis and digestion processes, thereby improving the efficiency and quality of DNA extraction. The elution volume used was 180 µl to ensure optimal DNA recovery. After the extraction, the quality and concentration of the DNA samples were measured using a spectrophotometer at wavelengths of 260/280 nm.

### Establishing a standard curve

The primer sequences used in this study were based on the research of Zhang et al. (2022)^40^ and were synthesized by Liuhe Huada Genomics Co., Ltd. (Beijing). These primers amplify a 146-bp fragment, and detailed information about the primer sequences is provided in Table 1.

**Table 1:** Primer pairs for mtDNA ctyb of *A. Schlegelii*.

Primer	Primer sequence (5’-3’)	5’modification	3’ modification
001-01-F	CTTCTCGGTCTCTGCTTAATCTCC		
001-01-R	GCGTGGAGGTTTCGGATTAG		
001-01-Probe	ACTTCCGACATTGCCAC	5’-FAM	3’-MGB

The full gene sequence was synthesized using the target sequence as a template, and the synthesized sequence was subsequently cloned and inserted into the PUC57 plasmid.

CTTCTCGGTCTCTGCTTAATCTCCCAACTTCTTACAGGACTATTTCTTGCCATGCACTATACTTCCGACATTGCCA; CAGCCTTTTCTTCCGTAGCTCACATCTGCCGAGATGTAAACTACGGATGACTAATCCGAAACCTCCACGC.

For the purpose of quantifying the target gene, quantitative polymerase chain reaction (qPCR) analysis was performed. Plasmids were used to prepare a series of tenfold dilutions ranging from *n*×10^9^ to *n*×10^3^ copies to construct a standard curve.

The PCR volume was 10 µL. The thermal cycling conditions included initial denaturation at 95 °C for 2 min, followed by 40 cycles of 95 °C for 10 s, 60 °C for 10 s, and 72 °C for 10 s. The detailed reagent volumes are provided in Table 2.

**Table 2:** Reaction system and amount of PCR.

Reaction system	Dosage(µL)
H_2_O	0.7 µl
2×PCR Mix	5 µl
Primer(up10 pM/µl)	0.5 µl
Primer(down10 pM/µl)	0.5 µl
Probe(down10 pM/µl)	0.25 µl
Template cDNA	3 µl
ROX	0.05 µl

**Fig. 1:**
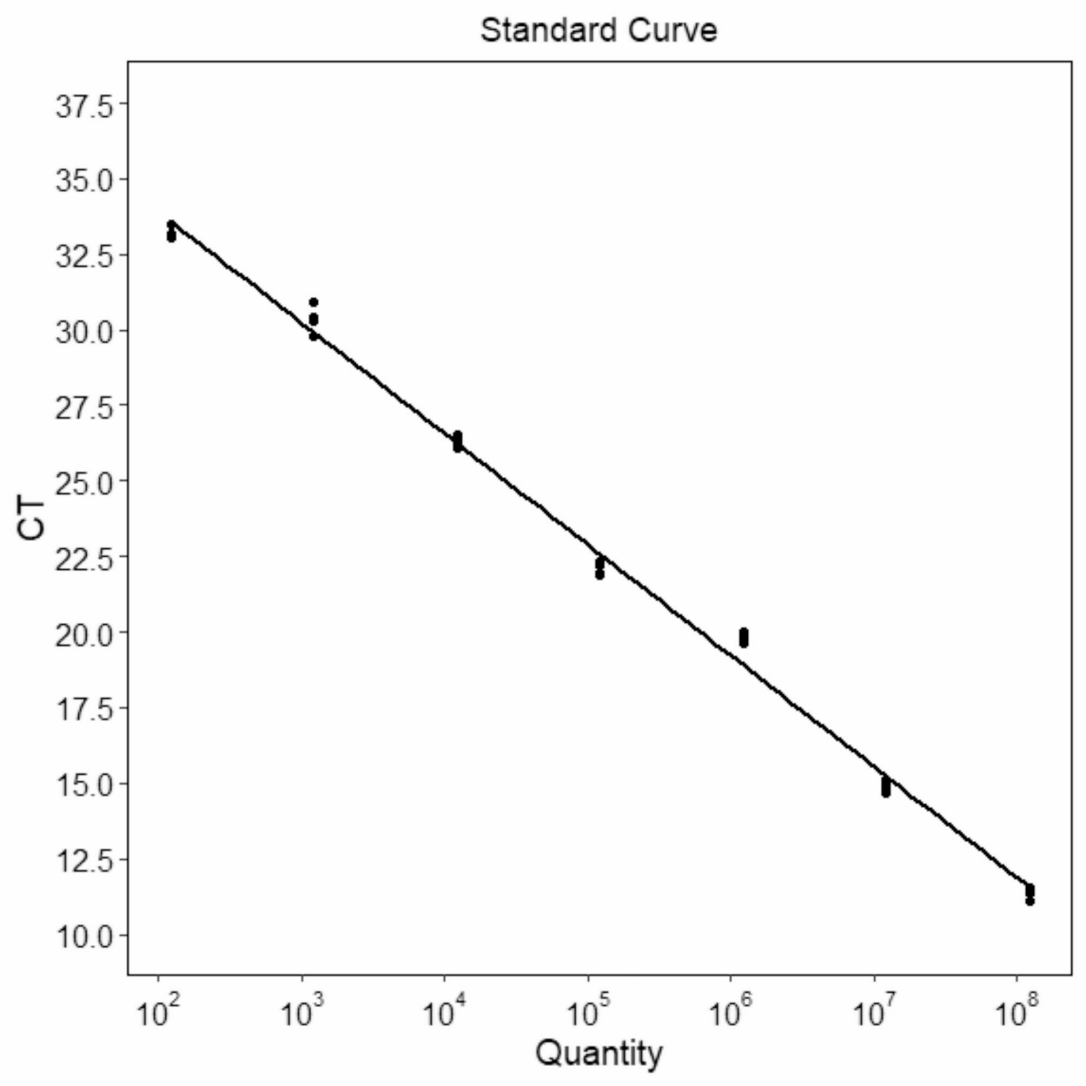
The standard curve of qPCR of the *A. schlegelii* Cytb gene.

A standard curve was established for the Cytb gene of *A. schlegelii* using qPCR technology. The curve was used to quantitatively analyze the variation in fluorescence values, with a slope (K value) of -3.671, an amplification efficiency of 87.249%, a correlation coefficient (R^2^) of 0.995, and a Y-intercept of 41.242, demonstrating a strong linear relationship (Fig. 1).

### Data analysis

Excel was used for the standardization of experimental data and R software version 4.0.4^41^ for data processing and visualization analysis. In terms of model selection, the performance of the models was evaluated by calculating the Akaike information criterion (AIC)^42^. The ‘stats’ and ‘mgcv’ packages in R were used to fit linear models (LM, both simple linear and univariate quadratic)^43^, generalized linear models (GLM)^44^, and generalized additive models (GAM, with Gaussian and Inverse Gaussian distributions)^45^ using the functions ‘lm()’, ‘glm()’, and ‘gam()’, respectively. These five models were designed to capture both linear and nonlinear relationships in the data, and nonlinear effects were handled using natural logarithms among other link functions.

### Statistical analysis

The statistical analysis was conducted using IBM SPSS Statistics software, version 25.0^46^. We employed a one-way ANOVA to assess the significance of differences among the groups.

## Results

### Influence of salinity on the eDNA concentration

**Fig. 2:**
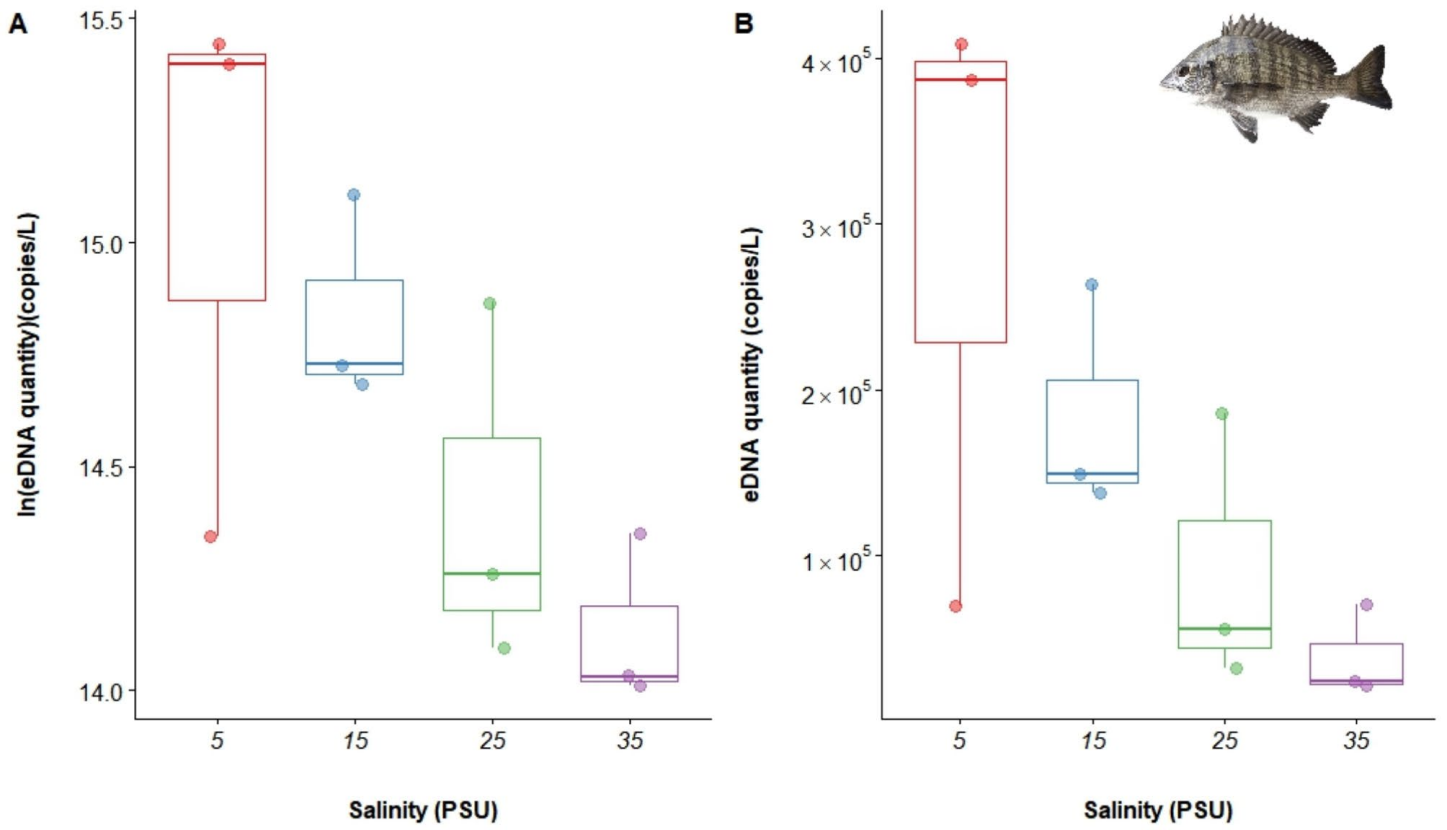
Box plot of the relationship between the number of eDNA copies and salinity in *A. schlegelii*.

The eDNA from *A. schlegelii* was undetectable in both the blank and negative controls. In aquaculture tanks with salinities of 5, 15, 25, and 35, the weighted average copy numbers of *A. schlegelii* eDNA were 8.43 × 10^4^ copies/L, 6.53 × 10^4^ copies/L, 4.54 × 10^4^ copies/L, and 3.21 × 10^4^ copies/L, respectively. This trend suggests a negative correlation between salinity and eDNA concentration (Fig. 2). A one-way ANOVA test was conducted to compare the eDNA concentrations across salinity groups (*P* = 0.096), which was close to the threshold for statistical significance, suggesting a trend towards significance with a larger sample size.

**Fig. 3:**
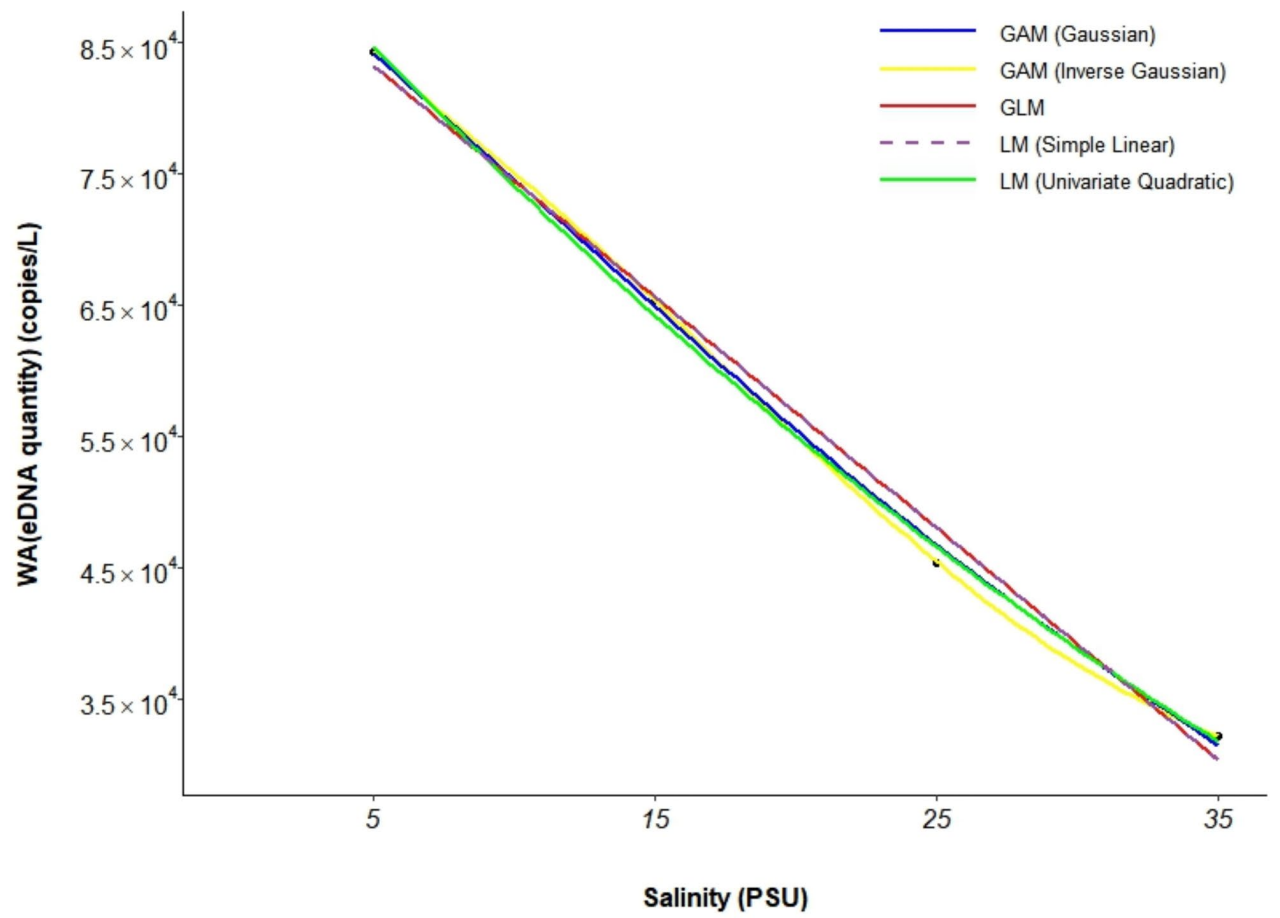
Multimodel fit of the relationship between eDNA and salinity in *A. schlegelii*.

A comparison of the AIC values of the five models (Table 3) revealed that the GAM (Inverse Gaussian) had the lowest AIC (30.026) and an R^2^ of 1.000, indicating an extremely high degree of fit. The *P* value was 0.0001, making it the best model for fitting the relationship between *A. schlegelii* eDNA concentration and salinity (Fig. 3; Fig. S1).

**Table 3:** The choice of model based on the AIC.

	GAM (Inverse Gaussian)	GAM (Gaussian)	GLM	LM (Simple Linear)	LM (Univariate Quadratic)
AIC	30.026	73.075	76.604	76.604	73.322
R^2^	1.000	0.996	0.990	0.990	0.995
*P* value	0.0001	0.042	0.004	0.004	0.043
Deviance explained	100%	99.90%	-	-	-

### Mechanism of eDNA release

**Fig. 4:**
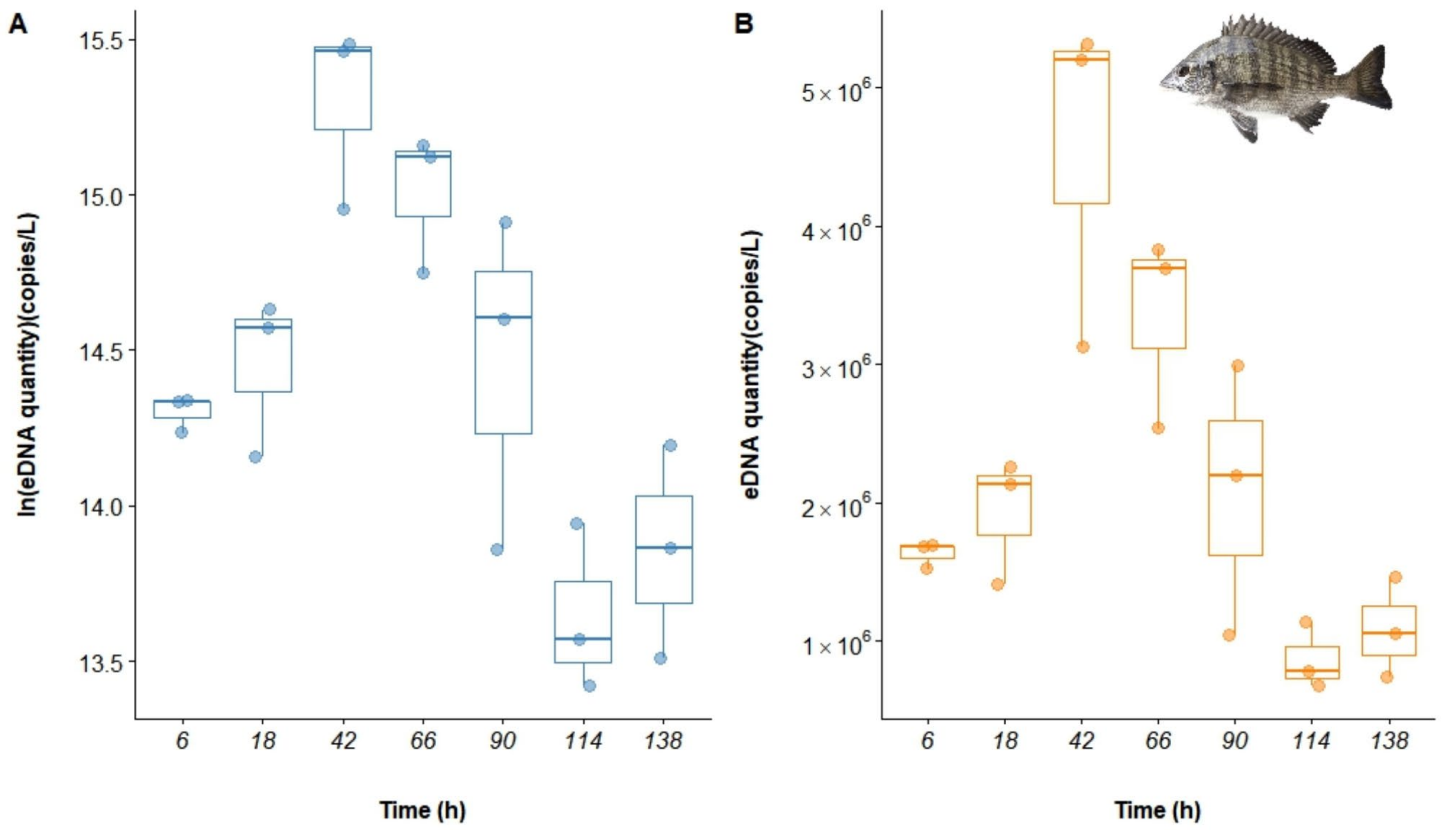
Box plot of *A. schlegelii* eDNA release rates.

The eDNA from *A. schlegelii* was undetectable in both the blank and negative controls. Analysis of the data indicated that the copy number of eDNA began to increase gradually after the experiment commenced, reaching a peak at 42 h, followed by a decrease and stabilization after 114 h (Fig. 4). Statistical analysis via one-way ANOVA confirmed significant changes in eDNA concentrations over time (*P* < 0.001).

**Fig. 5:**
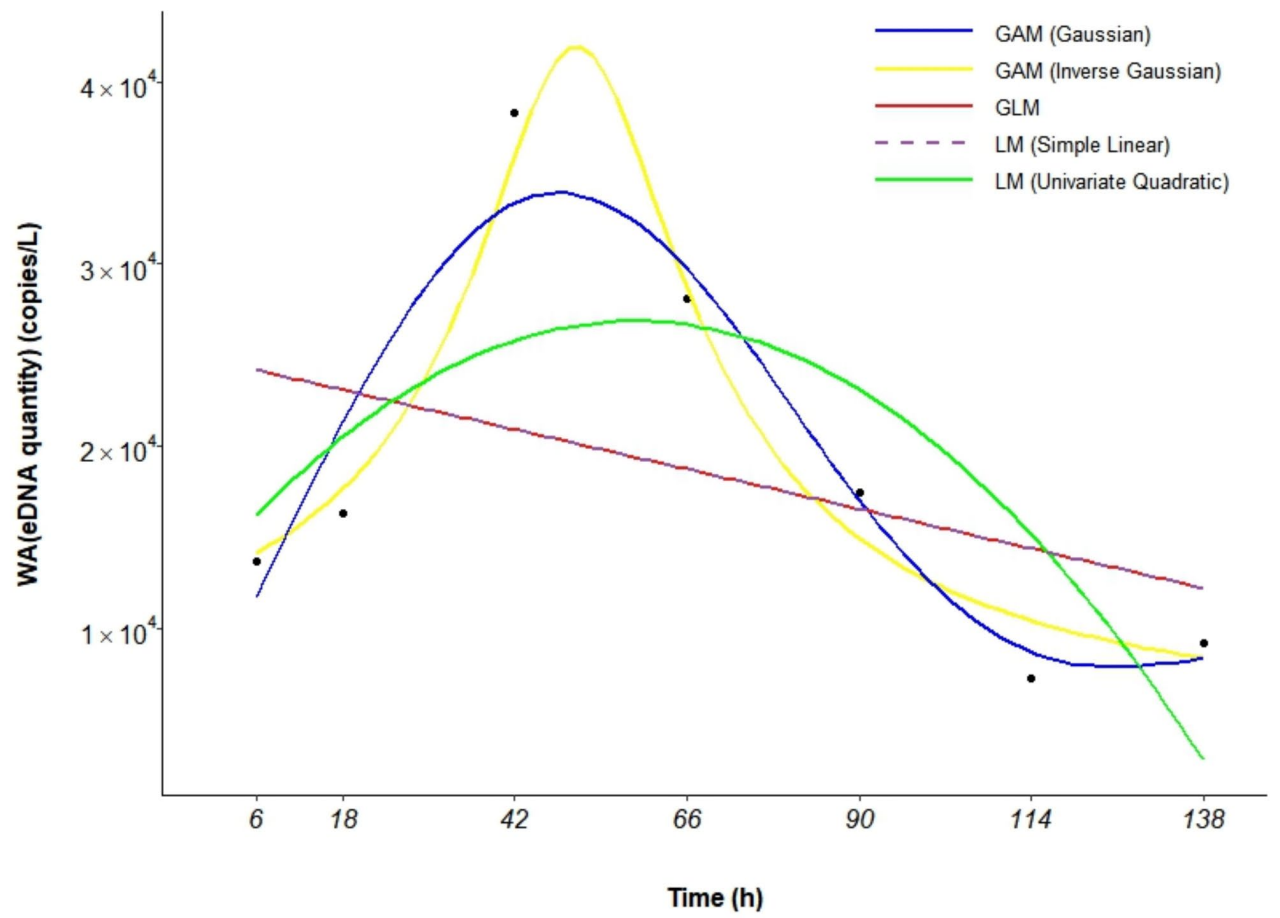
Multimodel fit of *A. schlegelii* eDNA release rates.

Among the five models compared (Table 4), the GAM (Gaussian) had the lowest AIC (141.377), with an R^2^ of 0.847 and a *P* value of 0.038, indicating a good fit and high explanatory power of 92%. This model was therefore identified as the optimal model for describing the dynamics of *A. schlegelii* eDNA release, accurately capturing the observed trends (Fig. 5; Fig. S2).

**Table 4:** The choice of model based on the AIC.

	GAM (Inverse Gaussian)	GAM (Gaussian)	GLM	LM (Simple Linear)	LM (Univariate Quadratic)
AIC	144.456	141.377	153.824	153.824	151.290
R^2^	0.922	0.847	-0.003	-0.003	0.344
*P* value	0.046	0.038	0.367	0.367	0.191
Deviance explained	86.00%	92.00%	-	-	-

### Mechanism of eDNA degradation

**Fig. 6:**
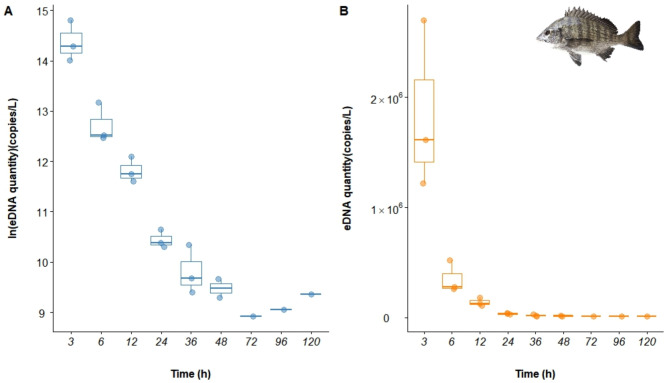
Box plot of *A. schlegelii* eDNA degradation rates.

The eDNA of *A. schlegelii* was undetectable in both the blank and negative controls. The data analysis revealed a negative correlation between the degradation rate of *A. schlegelii* eDNA and time. Within the first 6 h after removal of *A. schlegelii*, the eDNA concentration decreased rapidly. As time progressed, the copy number of eDNA in the water continued to decrease, slowing after 24 h and gradually stabilizing, approaching zero levels. Trace amounts of eDNA were still detectable in the water at 120 h (Fig. 6). Statistical analysis via one-way ANOVA confirmed significant changes in eDNA concentrations over time (*P* < 0.001).

**Fig. 7:**
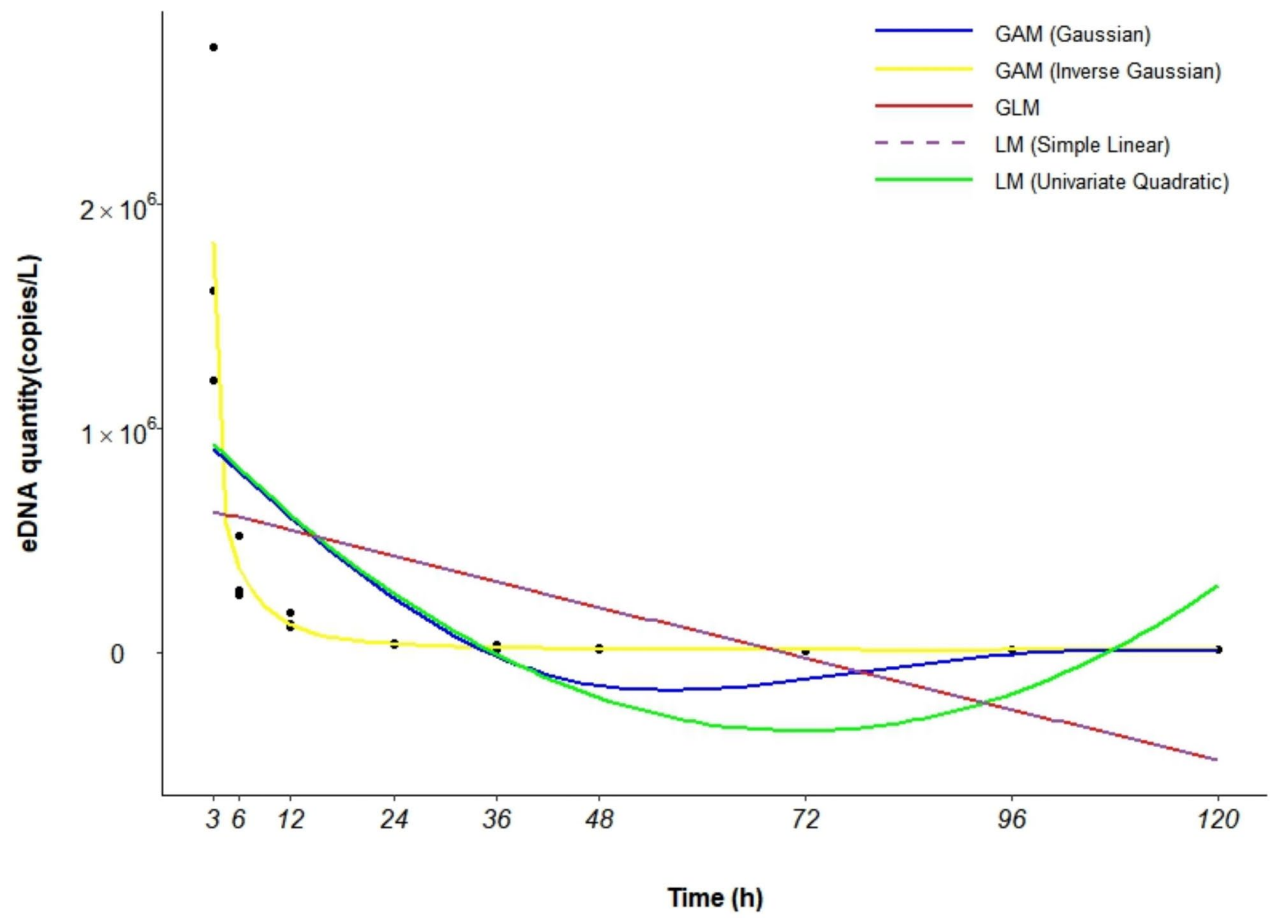
Multimodel fit of *A. schlegelii* eDNA degradation rates.

Among the five models compared (Table 5), the GAM (Inverse Gaussian) model showed the best fit for the eDNA degradation data, with the lowest AIC (480.644), an R^2^ of 0.842, and a *P* value of 0.000004, accounting for 94.80% of the variance. This model was therefore identified as the optimal for describing *A. schlegelii* eDNA degradation dynamics (Fig. 7; Fig. S3).

**Table 5:** The choice of model based on the AIC.

	GAM (Inverse Gaussian)	GAM (Gaussian)	GLM	LM (Simple Linear)	LM (Univariate Quadratic)
AIC	480.644	591.999	595.602	595.602	591.800
R^2^	0.842	0.349	0.150	0.150	0.327
*P* value	0.000004	0.026	0.051	0.051	0.013
Deviance explained	94.80%	42.40%	-	-	-

### The relationship between biomass and eDNA concentration

**Fig. 8:**
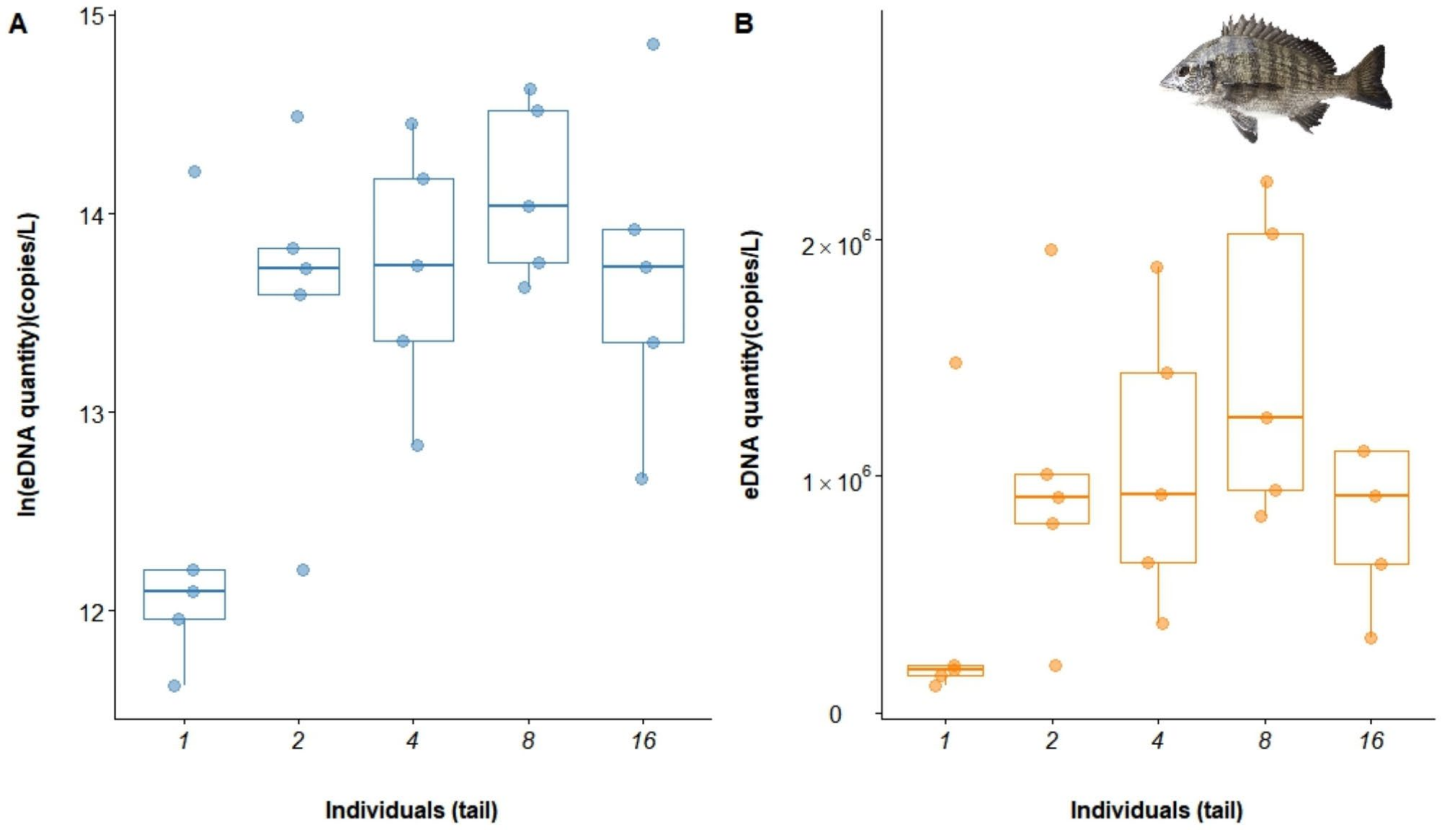
Box plot of the relationship between eDNA and biomass in *A. schlegelii*.

The eDNA of *A. schlegelii* was undetectable in both the blank and negative controls. The results indicated a significant correlation between *A. schlegelii* biomass and eDNA concentration. As the number of individuals increased from 1 to 2, with the average weight increasing from 0.13 kg to 0.24 kg, there was an observed increase in the eDNA concentration. With a further increase to 4 individuals, the average weight reached 0.52 kg, and the rate of increase in eDNA concentrations began to slow, approaching a plateau. When the number of fish increased to 8, with an average weight of 0.92 kg, the eDNA concentration showed an increase. However, when the number of fish reached 16, with an average weight of 1.81 kg, the eDNA concentration decreased (Fig. 8). A one-way ANOVA test was conducted to assess the significance of these variations, yielding a P-value of 0.264. Given the high P-value, we conclude that the differences observed are not statistically significant, possibly due to low replicate numbers. Further studies with increased replication may provide a clearer understanding of the relationship.

**Fig. 9:**
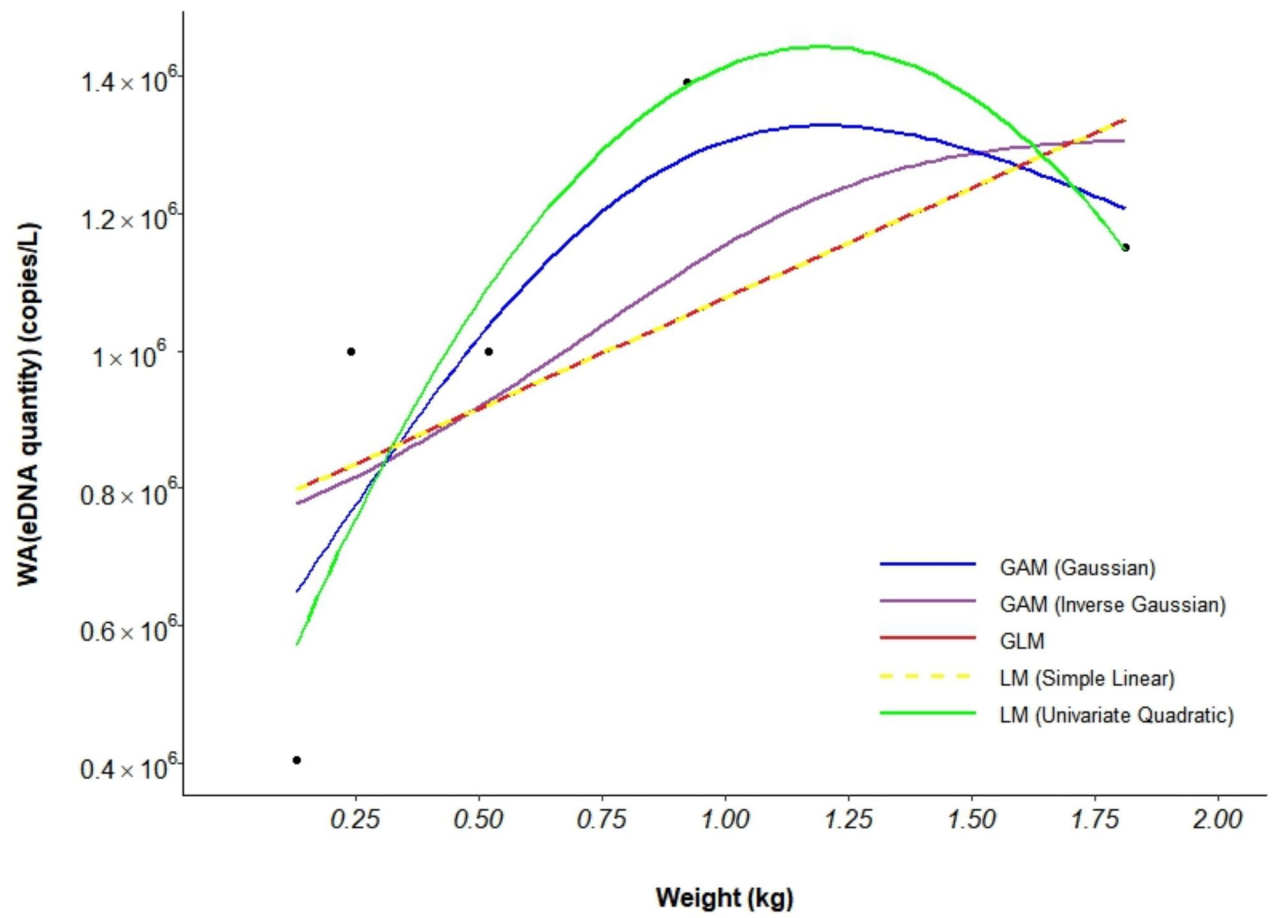
Multimodel fit of the relationship between eDNA and biomass in *A. schlegelii*.

Among the five models compared (Table 6), the LM (Univariate Quadratic Regression) model offered the best fit, with the lowest AIC value (140.934) and the highest R^2^ value (0.611), a *P* value of 0.195. Consequently, this study selects the LM (Univariate Quadratic Regression) as the optimal model, with the fitted curve equation: y = 988,511 + 436702x − 485308x^2^ (Fig. 9; Fig. S4).

**Table 6:** The choice of model based on the AIC.

	GAM (Inverse Gaussian)	GAM (Gaussian)	GLM	LM (Simple Linear)	LM (Univariate Quadratic)
AIC	148.4627	142.4207	144.882	144.882	140.934
R^2^	0.035	0.545	0.147	0.147	0.611
*P* value	0.437	0.271	0.284	0.284	0.195
Deviance explained	22.60%	75.10%	-	-	-

## Discussion

### Influence of salinity on the eDNA concentration

This study explored the relationship between the concentration of *A. schlegelii* eDNA and salinity. The results indicated a negative correlation, with eDNA concentrations decreasing as salinity increases. This finding aligns with previous studies and further corroborates the impact of salinity on eDNA stability. In high-salinity environments, the degradation of eDNA may accelerate, reducing its persistence in marine settings and decreasing its detectability. Schmelzle and Kinziger (2016) used a GLM to analyze eDNA samples from *Eucyclogobius newberryi* in lagoons and estuaries and noted a significant decrease in eDNA concentration with increasing salinity^47^. This association has also been supported by multiple other researchers^11,48,49^.

However, contrary findings have also been observed. For instance, Xin et al. (2022) studied eDNA released by *Acanthopagrus latus* using qPCR techniques and reported an increasing trend in eDNA concentration with increasing salinity, suggesting a potential positive correlation between eDNA concentration and salinity^27^. Similarly, Saito and Doi (2021) analyzed water samples from marine and pond environments and discovered slower degradation rates of eDNA under high-salinity conditions, which indicated prolonged persistence and a possible positive correlation between eDNA concentration and salinity^26^. Furthermore, Collins et al. (2018) analyzed data across different salinity gradients at marine sites, observing slower degradation rates at high-salinity sites and therefore higher concentrations of eDNA^50^.

In summary, while the majority of studies support a negative correlation between salinity and eDNA concentration, this general trend does not apply universally across all species and environmental conditions^51^. In fact, salinity can have varying or even opposite effects on the stability and detectability of eDNA, highlighting the importance of considering species-specific responses and environmental factors^51^. These differences underscore the need to consider the unique ecological traits and survival strategies of different species in response to environmental variables^52,53^. Additionally, other environmental factors, such as temperature, water chemistry, and light conditions, may also interact with salinity, further influencing eDNA behavior^35^. Therefore, to accurately assess and monitor the biomass and ecological status of aquatic organisms, it is essential to explore these complex interactions across a broader range of species and environmental settings to better understand how salinity affects the application of eDNA techniques.

### Investigating the release mechanisms of environmental DNA

Although eDNA technology is still in its nascent stages in the field of aquatic biomass estimation, its demonstrated potential underscores its critical importance for future research and application^54^. Among the key parameters for accurate biomass estimation is the eDNA release rate—the amount of DNA released by a target species per unit of time^55^. Current knowledge about eDNA release rates is insufficient, particularly regarding variations among different species, which has become a major bottleneck limiting the precision of this technology^8^. A precise understanding of the eDNA release rates of different species is crucial for enhancing the accuracy of biomass assessments and has practical implications for the conservation and management of aquatic biological resources^56^.

This study includes a detailed analysis of the eDNA release patterns of *A. schlegelii* at various time points, revealing that its eDNA concentration peaked at 42 h and stabilized after 114 h. This finding contrasts with findings from other studies, such as Minamoto et al. (2017), who investigated the eDNA release rates of *Chrysaora pacifica* and noted two peaks at 1 h and 8 h, followed by stabilization at 48 h^24^. Although the eDNA concentration dynamics of *A. schlegelii* differ from those of *C. pacifica*, these results also indicate potential species-specific variations in eDNA release patterns. Similarly, Maruyama et al. (2014) demonstrated that in *Lepomis macrochirus*, eDNA release rates are closely linked to the fish developmental stage, with adults releasing more eDNA than juveniles. This finding highlights how the developmental stage of a biological entity may significantly impact eDNA release rates^57^. Takahara et al. (2012) showed in their study of *Cyprinus carpio* L. in an aquarium setting that the eDNA concentrations in water containing three *C. carpio* L. peaked on the first day, whereas those in water containing a single *C. carpio* L. peaked on the second day, with eDNA concentrations stabilizing after six days. These results reveal the effects of individual numbers within the same species on eDNA concentration changes under specific conditions^25^. Furthermore, eDNA release rates are influenced not only by species, size, age, and physiological and behavioral characteristics^24^ but also by external environmental factors such as water temperature, pH, and the presence of predators^32,58,59^.

Therefore, future efforts must continue to explore the eDNA release patterns of different species and systematically consider the impacts of environmental factors to apply eDNA technology more accurately for aquatic biomass estimation and ecological research. Such in-depth studies will not only enhance our understanding of eDNA release processes in marine organisms but also promote the widespread use of eDNA as a tool for biological monitoring, providing valuable information for future environmental surveillance and ecological studies.

### Investigating the degradation mechanisms of environmental DNA

This study extensively investigated the degradation process of *A. schlegelii* eDNA over time and revealed that the eDNA concentration rapidly decreased within the first 24 h and remained at an extremely low level for the following 120 h. This degradation was categorized into two phases: rapid and slow. The results indicated that the persistence of *A. schlegelii* eDNA in marine environments is approximately five days. Previous studies have demonstrated significant variation in eDNA degradation rates among different species. For instance, Barnes et al. (2014) reported that *C. carpio* eDNA persists in water for approximately 4.2 days^60^. Dejean et al. (2011) noted a persistence of up to 14 days for *Acipenser baerii* and up to 25 days for *Rana catesbeiana* (*syn. Lithobates catesbeianus*)^61^, while Strickler et al. (2015) reported a persistence of 58 days for the latter^34^. Sassoubre et al. (2016) observed a persistence of 3–4 days for species such as *Engraulis mordax*, *Sardinops sagax*, and *Scomber japonicas*^55^. Piaggio et al. (2014) reported a persistence range of 2–7 days for *Python bivittatus*^62^, and Pilliod et al. (2014) reported a three-day persistence for *Dicamptodon aterrimus*^63^. Therefore, to deepen our understanding of the behavior of eDNA in aquatic environments, targeted studies on specific species are essential to enrich our knowledge base of eDNA degradation patterns, which is crucial for optimizing eDNA monitoring strategies and accurately interpreting eDNA data.

Moreover, once released into natural environments, eDNA undergoes a degradation process, breaking down into shorter fragments^64^. Previous studies have shown that even when eDNA persists in water for weeks, short fragments can still be detected, demonstrating a degree of stability^61,65^. Jo et al. (2017) compared the degradation rates of long (719 bp) and short (127 bp) eDNA fragments and reported that longer fragments degrade more rapidly, highlighting the importance of studying short fragments to enhance species detection rates^66^. In addition to fragment length, other factors that significantly influence the degradation rate of eDNA include temperature, microbial activity, and UV-B radiation. Higher temperatures enhance microbial metabolism and exonuclease activity, while extremely high temperatures can cause DNA denaturation, affecting degradation^67−69^. Microorganisms contribute to DNA breakdown through the production of exogenous nucleases^70^. UV-B radiation can cause photolytic DNA damage, which hinders PCR amplification^71^. Further studies are needed to explore the interactions of these factors and their specific impacts, especially in complex marine environments. Therefore, a deep understanding of eDNA degradation mechanisms is vital for applying eDNA technology in field surveys and biological monitoring.

### Relationship between biomass and eDNA concentration

This study investigated the relationship between the biomass of *A. schlegelii* and the eDNA concentration. The average weight of *A. schlegelii* increased from 0.13 kg to 0.92 kg, showing a positive correlation with increasing eDNA concentration. Similarly, Karlsson et al. (2022) assessed the biomass and eDNA concentration of *Esox lucius* in aquarium conditions and reported a strong positive correlation between the two parameters^72^. Xin et al. (2022) supported this relationship for *A. latus*^27^, and Takahara et al. (2012) also reported a positive correlation between eDNA concentration and biomass in various aquatic environments for *C. carpio* L^25^. Numerous studies have corroborated these findings^65,73,74^, underscoring the significance of using eDNA techniques for assessing fish biomass and distribution.

However, when the average weight of *A. schlegelii* reached 1.81 kg, the eDNA concentration did not increase but instead decreased, indicating a negative correlation. This outcome contrasts with previous findings, but a deeper analysis suggests that this phenomenon may result from a combination of individual differences, environmental factors, and species specificity. First, physiological factors such as size, age, health status, and metabolic activity can vary significantly among individuals within the same species, potentially leading to different eDNA release rates and thereby influencing the relationship between eDNA concentration and biomass^75,76^. Second, the relationship between eDNA concentration and fish biomass may also be affected by environmental factors, such as light intensity, UV radiation, and microbial activity, which can alter eDNA concentrations even under constant biomass conditions^66,73,77^. Additionally, species specificity cannot be overlooked. Different species may have distinct eDNA release and degradation mechanisms, suggesting that the relationship between eDNA concentration and biomass could vary by species. Some species may release eDNA more rapidly but with less stability, while others may do the opposite^78^. Further research has indicated that under conditions of high water temperatures and large biomass, the degradation rate of eDNA accelerates. These complex interactions significantly impact eDNA concentration and stability^32^, not only elucidating the observed decrease in eDNA concentration with increased *A. schlegelii* biomass but also providing a plausible explanation for the variability in eDNA concentration across parallel groups.

Therefore, this study posits that the complex relationship between *A. schlegelii* biomass and eDNA concentration is likely influenced by multiple factors. Spatial constraints may intensify individual stress, affecting metabolic rates and eDNA release rates. As biomass increases, the rate of eDNA degradation in the water may accelerate, particularly in aquaculture environments where microbial activity is enhanced, potentially adversely affecting eDNA stability. Furthermore, changes in water quality conditions, such as temperature, pH, and dissolved oxygen levels, can also impact the stability and detectability of eDNA. Future research should explore how these factors individually and collectively affect the release, distribution, and degradation of eDNA. By thoroughly considering these complex interactions, a more robust and precise theoretical foundation can be provided for the application of eDNA techniques in biomass assessment.

## Conclusion

In this study, experiments on the aquaculture of *A. schlegelii* were conducted, and different experimental groups were established to investigate the mechanisms of eDNA release and degradation in *A. schlegelii*. The relationships between eDNA concentration and biomass and salinity were explored. Through modeling, the variations in eDNA under different conditions were fitted, clarifying the patterns of eDNA changes in *A. schlegelii*. This initial investigation of the fluctuations in eDNA concentrations in water under various conditions lays the foundation for the future development of eDNA-based quantitative detection techniques for estimating *A. schlegelii* biomass in natural marine areas. This study also provides a reference for further research on quantitative detection techniques for eDNA in other marine fish species.

This study used eDNA technology to evaluate *A. schlegelii* biomass and is still exploratory, with many experimental limitations. Environmental factors such as pH, light intensity, and temperature influence *A. schlegelii* eDNA concentrations. The interactions among these factors in complex marine environments are not yet fully understood, necessitating further research to refine eDNA quantification methods for this species. When studying the impact of biomass on the eDNA concentration in *A. schlegelii*, research should extend beyond mere counts and sizes to include growth stages, body size, and maturity. This comprehensive approach will enhance the accuracy of eDNA technology applications in real marine ecosystems. Current research has not fully captured the dynamics of *A. schlegelii* eDNA release and degradation across various environments. Considering interspecies differences, environmental diversity, and seasonal variations, further in-depth studies are required to explore eDNA dynamics in different contexts. Current eDNA methodologies lack standardization, making it difficult to compare results across different studies and complicating research efforts. There is an urgent need to develop repeatable and comparable standard procedures to ensure the accuracy and consistency of research findings. Moreover, large-scale field validation of eDNA biomass assessment techniques is crucial for verifying their effectiveness across various habitats and species, ensuring their reliability and accuracy in practical applications.

## Electronic supplementary material

Below is the link to the electronic supplementary material.

Supplementary Material 1

## Data Availability

The datasets used and analysed during the current study available from the corresponding author on reasonable request.

## References

[1] Huang, S. L., Dai, X. J. & Chen, Q. Current situation and existing problems of aquatic species enhancement and releasing in Shanghai water area. *Chin. Fish. Econ.* **04**, 79–87 (2009).

[2] Bell, J. D., Leber, K. M., Blankenship, H. L., Loneragan, N. R. & Masuda, R. A new era for restocking, stock enhancement and sea ranching of coastal fisheries resources. *Rev. Fish. Sci.* **16**, 1–9 (2008).

[3] Zhang, M. et al. Microsatellite-marker-based evaluation of stock enhancement for Kuruma Prawn *Pernaeus japonicus* in Beibu Gulf, South China Sea. *Fishes* **8**, 568 (2023).

[4] Shaw, J. L. A. et al. Comparison of environmental DNA metabarcoding and conventional fish survey methods in a river system. *Biol. Conserv.* **197**, 131–138 (2016).

[5] Evans, N. T., Shirey, P. D., Wieringa, J. G., Mahon, A. R. & Lamberti, G. A. Comparative cost and effort of fish distribution detection via environmental DNA analysis and electrofishing. *Fisheries* **42**, 90–99 (2017).

[6] Yamamoto, S. et al. Environmental DNA as a snapshot of fish distribution: A case study of Japanese jack mackerel in Maizuru Bay, Sea of Japan. *PLoS One* **11**, e0149786 (2016). 26933889 10.1371/journal.pone.0149786 PMC4775019

[7] Wang, S. P. et al. Methodology of fish eDNA and its applications in ecology and environment. *Sci. Total Environ.* **755**, 142622 (2021). 33059148 10.1016/j.scitotenv.2020.142622

[8] Thomsen, P. F. & Willerslev, E. Environmental DNA-An emerging tool in conservation for monitoring past and present biodiversity. *Biol. Conserv.* **183**, 4–18 (2015).

[9] Harrison, J. B., Sunday, J. M. & Rogers, S. M. Predicting the fate of eDNA in the environment and implications for studying biodiversity. *P Roy Soc. B-Biol Sci.* **286**, 20191409 (2019). 10.1098/rspb.2019.1409 PMC6892050 31744434

[10] Taberlet, P., Coissac, E., Hajibabaei, M., Rieseberg, L. H. & Environmental, D. N. A. *Mol. Ecol.* **21** (2012). 10.1111/j.1365-294X.2012.05542.x 22486819

[11] Foote, A. D. et al. Investigating the potential use of environmental DNA (eDNA) for genetic monitoring of marine mammals. *PLoS One* **7**, e41781 (2012). 22952587 10.1371/journal.pone.0041781 PMC3430683

[12] Jerde, C. L., Mahon, A. R., Chadderton, W. L. & Lodge, D. M. Sight-unseen detection of rare aquatic species using environmental DNA. *Conserv. Lett.* **4**, 150–157 (2011).

[13] Laramie, M. B., Pilliod, D. S. & Goldberg, C. S. Characterizing the distribution of an endangered salmonid using environmental DNA analysis. *Biol. Conserv.* **183**, 29–37 (2015).

[14] Robinson, C. V., Webster, T. M. U., Cable, J., James, J. & Consuegra, S. Simultaneous detection of invasive signal crayfish, endangered white-clawed crayfish and the crayfish plague pathogen using environmental DNA. *Biol. Conserv.* **222**, 241–252 (2018).

[15] Balasingham, K. D., Walter, R. P., Mandrak, N. E. & Heath, D. D. Environmental DNA detection of rare and invasive fish species in two great lakes tributaries. *Mol. Ecol.* **27**, 112–127 (2018). 29087006 10.1111/mec.14395

[16] Dejean, T. et al. Improved detection of an alien invasive species through environmental DNA barcoding: The example of the American bullfrog *Lithobates catesbeianus*. *Ecol. Appl.* **49**, 953–959 (2012).

[17] Mahon, A. R. et al. Validation of eDNA surveillance sensitivity for detection of Asian carps in controlled and field experiments. *PLoS One* **8**, e58316 (2013). 23472178 10.1371/journal.pone.0058316 PMC3589332

[18] Kim, P., Kim, D., Yoon, T. J. & Shin, S. Early detection of marine invasive species, *Bugula neritina* (Bryozoa: Cheilostomatida), using species-specific primers and environmental DNA analysis in Korea. *Mar. Environ. Res.* **139**, 1–10 (2018). 29747863 10.1016/j.marenvres.2018.04.015

[19] Tréguier, A. et al. Environmental DNA surveillance for invertebrate species: Advantages and technical limitations to detect invasive crayfish *Procambarus clarkii* in freshwater ponds. *J. Appl. Ecol.* **51**, 871–879 (2014).

[20] Wilcox, T. M. et al. Understanding environmental DNA detection probabilities: A case study using a stream-dwelling char *Salvelinus fontinalis*. *Biol. Conserv.* **194**, 209–216 (2016).

[21] Minshall, G. W., Thomas, S. A., Newbold, J. D., Monaghan, M. T. & Cushing, C. E. Physical factors influencing fine organic particle transport and deposition in streams. *J. N. Am. Benthol Soc.* **19**, 1–16 (2000).

[22] Rice, C. J., Larson, E. R. & Taylor, C. A. Environmental DNA detects a rare large river crayfish but with little relation to local abundance. *Freshw. Biol.* **63**, 443–455 (2018).

[23] Stoeckle, B. C., Kuehn, R. & Geist, J. Environmental DNA as a monitoring tool for the endangered freshwater pearl mussel (*Margaritifera margaritifera* L.): A substitute for classical monitoring approaches? *Aquat. Conserv. Mar. Freshw. Ecosyst.* **26**, 1120–1129 (2015).

[24] Minamoto, T. et al. Environmental DNA reflects spatial and temporal jellyfish distribution. *PLoS One* **12**, e0173073 (2017). 28245277 10.1371/journal.pone.0173073 PMC5330514

[25] Takahara, T., Minamoto, T., Yamanaka, H., Doi, H. & Kawabata Z. I. Estimation of fish biomass using environmental DNA. *PLoS One* **7**, 1–8 (2012). 10.1371/journal.pone.0035868 PMC3338542 22563411

[26] Saito, T. & Doi, H. Degradation modeling of water environmental DNA: Experiments on multiple DNA sources in pond and seawater. *Environ. DNA* **3**, 850–860 (2021).

[27] Xin, Y. et al. Optimal conditions to quantify the relationship between eDNA concentration and biomass in *Acanthopagrus latus*. *Water* **14**, 3521 (2022).

[28] Coulter, D. P. et al. Nonlinear relationship between silver carp density and their eDNA concentration in a large river. *PLoS One* **14**, e0218823 (2019). 31242242 10.1371/journal.pone.0218823 PMC6594630

[29] Andruszkiewicz, E. A. et al. Modeling environmental DNA transport in the coastal ocean using Lagrangian particle tracking. *Front. Plant. Sci.* **6**, 477 (2019).

[30] Hansen, B. K., Bekkevold, D., Clausen, L. W. & Nielsen, E. E. The sceptical optimist: Challenges and perspectives for the application of environmental DNA in marine fisheries. *Fish. Fish.* **19**, 751–768 (2018).

[31] Doi, H. et al. Use of droplet digital PCR for estimation of fish abundance and biomass in environmental DNA surveys. *PLoS One* **10**, e0122763 (2015). 25799582 10.1371/journal.pone.0122763 PMC4370432

[32] Jo, T., Murakami, H., Yamamoto, S., Masuda, R. & Minamoto, T. Effect of water temperature and fish biomass on environmental DNA shedding, degradation, and size distribution. *Ecol. Evol.* **9**, 1135–1146 (2019). 30805147 10.1002/ece3.4802 PMC6374661

[33] Joseph, C., Faiq, M. E., Li, Z. & Chen, G. Persistence and degradation dynamics of eDNA affected by environmental factors in aquatic ecosystems. *Hydrobiologia* **849**, 4119–4133 (2022).

[34] Strickler, K. M., Fremier, A. K. & Goldberg, C. S. Quantifying effects of UV-B, temperature, and pH on eDNA degradation in aquatic microcosms. *Biol. Conserv.* **183**, 85–92 (2015).

[35] Takahara, T., Ikebuchi, T., Doi, H. & Minamoto, T. Using environmental DNA to estimate the seasonal distribution and habitat preferences of a Japanese basket clam in Lake Shinji, Japan. *Estuar. Coast Shelf Sci.* **221**, 15–20 (2019).

[36] Law, C. S. & de Sadovy, Y. Age and growth of black seabream *Acanthopagrus schlegelii* (Sparidae) in Hong Kong and adjacent waters of the northern South China Sea. *J. Fish. Biol.* **93**, 382–390 (2018). 30069882 10.1111/jfb.13774

[37] Wu, R. X., Liu, J., Fan, J. R. & Zhao, Y. J. A review on the nomenclature and taxonomic status of the black porgy, *Acanthopagrus Schlegelii* (Perciformes: Sparidae). *Mar. Sci.* **35**, 117–119 (2011).

[38] Gonzalez, E. B., Umino, T. & Nagasawa, K. Stock enhancement programme for black sea bream, *Acanthopagrus schlegelii* (Bleeker), in Hiroshima Bay, Japan: A review. *Aquacult. Res.* **39**, 1307–1315 (2008).

[39] Shan, B. et al. Parentage determination of black sea bream (*Acanthopagrus schlegelii*) for stock enhancement: Effectiveness and loss of genetic variation. *Acta Oceanol. Sin*. **40**, 41–49 (2021).

[40] Zhang, H., Zhou, Y., Zhang, H., Gao, T. & Wang, X. Fishery resource monitoring of the East China Sea via environmental DNA approach: A case study using black sea bream (*Acanthopagrus schlegelii*). *Front. Mar. Sci.* **9**, 848950 (2022).

[41] R Core Team. R: A language and environment for statistical computing; version 4.2.1 (2022).

[42] Akaike, H. A new look at the statistical model identification. *IEEE Trans. Autom. Control*. **19**, 716–723 (1974).

[43] Draper, N. R. & Smith, H. *Applied Regression Analysis* Vol. 326 (Wiley, 1998).

[44] McCullagh, P. *Generalized Linear Models* (Routledge, 2019).

[45] Wood, S. N. & Chapman Generalized additive models: An introduction with R. and Hall/CRC (2017).

[46] IBM & Corp *IBM SPSS Statistics for Windows (Version 25.0)* (IBM Corp, 2017).

[47] Schmelzle, M. C. & Kinziger, A. P. Using occupancy modelling to compare environmental DNA to traditional field methods for regional-scale monitoring of an endangered aquatic species. *Mol. Ecol. Resour.* **16**, 895–908 (2016). 26677162 10.1111/1755-0998.12501

[48] Thomsen, P. F. et al. Detection of a diverse marine fish fauna using environmental DNA from seawater samples. *PLoS One* **7**, e41732 (2012). 22952584 10.1371/journal.pone.0041732 PMC3430657

[49] Lamb, P. D., Fonseca, V. G., Maxwell, D. L. & Nnanatu, C. C. Systematic review and meta-analysis: Water type and temperature affect environmental DNA decay. *Mol. Ecol. Resour.* **22**, 2494–2505 (2022). 35510730 10.1111/1755-0998.13627 PMC9541873

[50] Collins, R. A. et al. Persistence of environmental DNA in marine systems. *Commun. Biol.* **1**, 1–11 (2018). 30417122 10.1038/s42003-018-0192-6 PMC6218555

[51] Zhang, J. et al. Species identification and biomass assessment of Gnathanodon speciosus based on environmental DNA technology. *Ecol. Indic.* **160**, 111821 (2024).

[52] Afzali, S. F. et al. Comparing environmental metabarcoding and trawling survey of demersal fish communities in the Gulf of St. Lawrence, Canada. *Environ. DNA* **3**, 22–42 (2021).

[53] Dunn, N., Priestley, V., Herraiz, A., Arnold, R. & Savolainen, V. Behavior and season affect crayfish detection and density inference using environmental DNA. *Ecol. Evol.* **7**, 7777–7785 (2017). 29043033 10.1002/ece3.3316 PMC5632632

[54] Tsuji, S. et al. Quantitative environmental DNA metabarcoding shows high potential as a novel approach to quantitatively assess fish community. *Sci. Rep.* **12**, 21524 (2022). 36513686 10.1038/s41598-022-25274-3 PMC9747787

[55] Sassoubre, L. M., Yamahara, K. M., Gardner, L. D., Block, B. A. & Boehm, A. B. Quantification of environmental DNA (eDNA) shedding and decay rates for three marine fish. *Environ. Sci. Technol.* **50**, 1045–10464 (2016). 10.1021/acs.est.6b03114 27580258

[56] Turner, C. R., Uy, K. L. & Everhart, R. C. Fish environmental DNA is more concentrated in aquatic sediments than surface water. *Biol. Conserv.* **183**, 93–102 (2015).

[57] Maruyama, A., Nakamura, K., Yamanaka, H., Kondoh, M. & Minamoto, T. The release rate of environmental DNA from juvenile and adult fish. *PLoS One* **9**, e114639 (2014). 25479160 10.1371/journal.pone.0114639 PMC4257714

[58] Klymus, K. E., Richter, C. A., Chapman, D. C. & Paukert, C. Quantification of eDNA shedding rates from invasive bighead carp *Hypophthalmichthys nobilis* and silver carp *Hypophthalmichthys molitrix*. *Biol. Conserv.* **183**, 77–84 (2015).

[59] Andruszkiewicz Allan, E., Zhang, W. G., Lavery, C., Govindarajan, F. & A. & Environmental DNA shedding and decay rates from diverse animal forms and thermal regimes. *Environ. DNA* **3**, 492–514 (2021).

[60] Barnes, M. A. et al. Environmental conditions influence eDNA persistence in aquatic systems. *Environ. Sci. Technol.* **48**, 1819–1827 (2014). 24422450 10.1021/es404734p

[61] Dejean, T. et al. Persistence of environmental DNA in freshwater ecosystems. *PLoS One* **6**, e23398 (2011). 21858099 10.1371/journal.pone.0023398 PMC3152572

[62] Piaggio, A. J. et al. Detecting an elusive invasive species: A diagnostic PCR to detect Burmese python in Florida waters and an assessment of persistence of environmental DNA. *Mol. Ecol. Resour.* **14**, 374–380 (2014). 24119154 10.1111/1755-0998.12180

[63] Pilliod, D. S., Goldberg, C. S., Arkle, R. S. & Waits, L. P. Factors infuencing detection of eDNA from a stream-dwelling amphibian. *Mol. Ecol. Resour.* **14**, 109–116 (2014). 24034561 10.1111/1755-0998.12159

[64] Bista, I. et al. Annual time-series analysis of aqueous eDNA reveals ecologically relevant dynamics of lake ecosystem biodiversity. *Nat. Commun.* **8**, 14087 (2017). 28098255 10.1038/ncomms14087 PMC5253663

[65] Goldberg, C. S., Sepulveda, A., Ray, A., Baumgardt, J. & Waits, L. P. Environmental DNA as a new method for early detection of New Zealand mudsnails (*Potamopyrgus antipodarum*). *Freshw. Sci.* **32**, 792–800 (2013).

[66] Jo, T. et al. Rapid degradation of longer DNA fragments enables the improved estimation of distribution and biomass using environmental DNA. *Mol. Ecol. Resour.* **17**, e25–e33 (2017). 28449215 10.1111/1755-0998.12685

[67] Fu, X. H., Wang, L., Le, Y. Q. & Hu, J. J. Persistence and renaturation efficiency of thermally treated waste recombinant DNA in defined aquatic microcosms. *J. Environ. Health A* **47**, 1975–1983 (2012). 10.1080/10934529.2012.695260 22870994

[68] Poté, J., Ackermann, R. & Wildi, W. Plant leaf mass loss and DNA release in freshwater sediments. *Ecotoxicol. Environ. Saf.* **72**, 1378–1383 (2009). 19419763 10.1016/j.ecoenv.2009.04.010

[69] Zhu, B. Degradation of plasmid and plant DNA in water microcosms monitored by natural transformation and real-time polymerase chain reaction (PCR). *Water Res.* **40**, 3231–3238 (2006). 16945402 10.1016/j.watres.2006.06.040

[70] Lindahl, T. Instability and decay of the primary structure of DNA. *Nature* **362**, 709–715 (1993). 8469282 10.1038/362709a0

[71] Häder, D. P., Kumar, H. D., Smith, R. C. & Worrest, R. C. Aquatic ecosystems: Effects of solar ultraviolet radiation and interactions with other climatic change factors. *Photochem. Photobiol Sci.* **2**, 39–50 (2003). 12659538 10.1039/b211160h

[72] Karlsson, E. et al. Strong positive relationships between eDNA concentrations and biomass in juvenile and adult pike (*Esox lucius*) under controlled conditions: Implications for monitoring. *Environ. DNA* **4**, 881–893 (2022).

[73] Pilliod, D. S., Goldberg, C. S., Arkle, R. S. & Waits, L. P. Estimating occupancy and abundance of stream amphibians using environmental DNA from filtered water samples. *Can. J. Fish. Aquat. Sci.* **70**, 1123–1130 (2013).

[74] Pont, D. et al. Environmental DNA reveals quantitative patterns of fish biodiversity in large rivers despite its downstream transportation. *Sci. Rep.* **8**, 10361 (2018). 29991759 10.1038/s41598-018-28424-8 PMC6039509

[75] Yates, M. C., Cristescu, M. E. & Derry, A. M. Integrating physiology and environmental dynamics to operationalize environmental DNA (eDNA) as a means to monitor freshwater macro-organism abundance. *Mol. Ecol.* **30**, 6531–6550 (2021). 34592014 10.1111/mec.16202

[76] Rourke, M. L. et al. Environmental DNA (eDNA) as a tool for assessing fish biomass: A review of approaches and future considerations for resource surveys. *Environ. DNA* **4**, 9–33 (2022).

[77] Lacoursiere-Roussel, A., Rosabal, M. & Bernatchez, L. Estimating fish abundance and biomass from eDNA concentrations: Variability among capture methods and environmental conditions. *Mol. Ecol. Resour.* **16**, 1401–1414 (2016). 26946353 10.1111/1755-0998.12522

[78] Barnes, M. A. & Turner, C. R. The ecology of environmental DNA and implications for conservation genetics. *Conserv. Genet.* **17**, 1–17 (2016).

