# Supplementary material for: Experimental assessment of Acanthopagrus schlegelii biomass based on environmental DNA technology
Source: Sci Rep. 2024 Dec 30;14:32029. doi: 10.1038/s41598-024-83590-2 (PMC11686245; doi:10.1038/s41598-024-83590-2)
Supplement: Supplementary file 1 — Supplementary Material 1 [file 41598_2024_83590_MOESM1_ESM.docx]

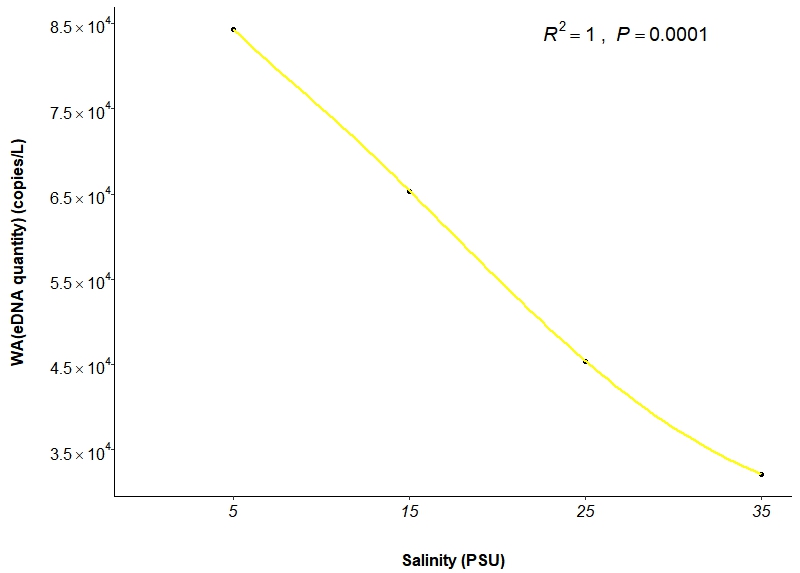


**Fig.** S1**.** GAM (inverse Gaussian) fit of the relationship between eDNA and salinity in *A. schlegelii*

*
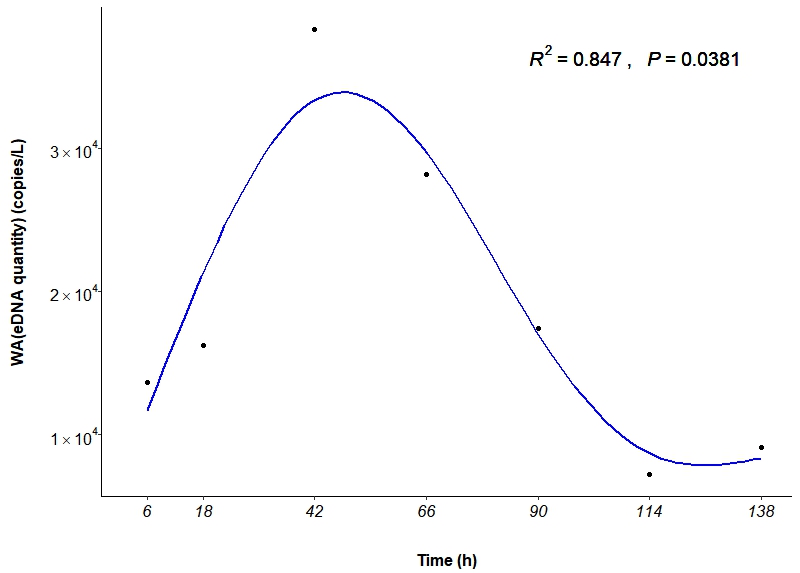
*

**Fig. S2.** GAM (Gaussian) fit of *A. schlegelii* eDNA release rates


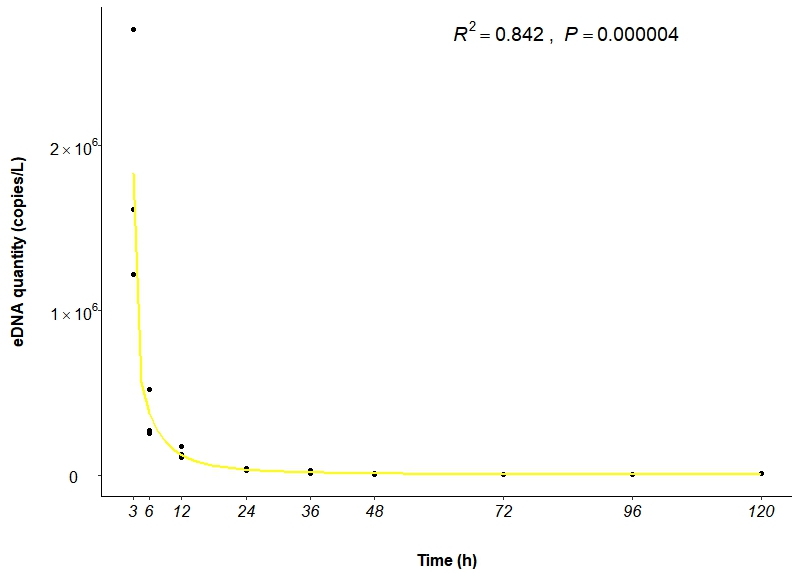


**Fig. S3.** GAM (Inverse Gaussian) fit of *A. schlegelii* eDNA degradation rates


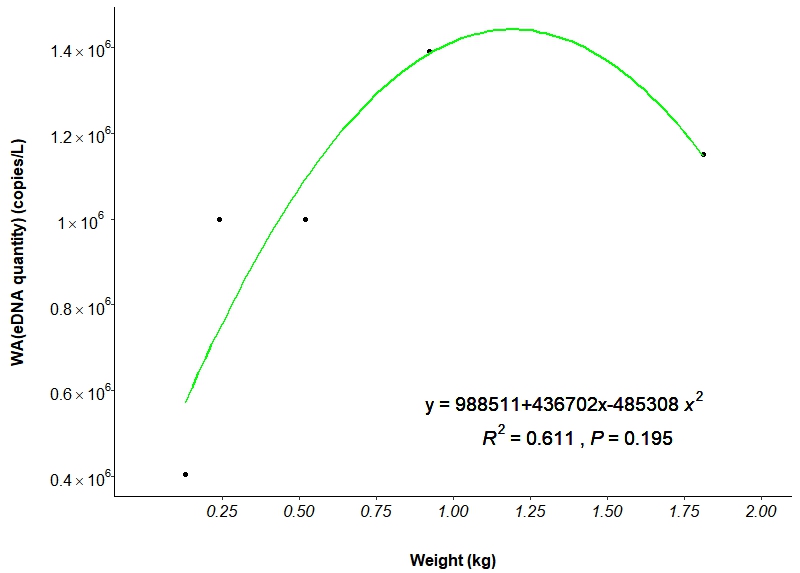


**Fig. S4.** LM fit of the relationship between eDNA and biomass in *A. schlegelii*
